# Evaluation of Cardiovascular Risk in Hidradenitis Suppurativa Patients Using Heart Rate Variability (HRV) Analysis

**DOI:** 10.1155/2020/1321782

**Published:** 2020-06-27

**Authors:** Nevena Skroza, Alessandra Mambrin, Ilaria Proietti, Veronica Balduzzi, Nicoletta Bernardini, Anna Marchesiello, Simone Michelini, Ersilia Tolino, Salvatore Volpe, Patrizia Maddalena, Antonella Ciaramella, Gianfranco Raimondi, Concetta Potenza

**Affiliations:** ^1^Department of Medical-Surgical Sciences and Biotechnologies, Dermatology Unit “Daniele Innocenzi”, Sapienza University of Rome, Polo Pontino, Italy; ^2^Department of Internal Medicine, Sapienza University, Rome, Italy

## Abstract

Hidradenitis suppurativa (HS) is a chronic inflammatory skin disease associated with elevated prevalence of comorbidities, especially metabolic and cardiovascular diseases. We used a tool called Heart Rate Variability (HRV) in order to assess the correlation between HS and alterations of the sympathetic-vagal equilibrium in the autonomic cardiovascular regulation system. We found increased sympathetic activity, associated with a higher risk of cardiovascular disease. HS, according to our results, is an independent cardiovascular risk factor.

## 1. Introduction

Hidradenitis suppurativa (HS) is a chronic inflammatory skin disease characterized by recurrent skin nodules, abscesses, and draining sinus tracts affecting primarily the apocrine gland-rich regions of the skin, especially the axillae and groin [1, 2].

HS has a chronic evolution with unpredictable periodic exacerbations. The lesions show a tendency to turn into fistulous tunnels, draining externally a serum-purulent material healing with the formation of retracting scars. Prevalence is estimated from <1% to 4% [3, 4], and the incidence is 11.4 cases per 100,000 patients, which is steadily increasing. Previous studies revealed a female predominance of 3 : 1 in the general population [5, 6]. Genetic factors play a fundamental role in the pathogenesis of HS, and one-third of patients report a positive family history of the disease. However, although the HS pathogenetic mechanism is still unknown, follicular hyperkeratinization probably characterizes the first step of the inflammatory process [7]. Histological examination revealed follicular hyperkeratosis and lymphocytic perifollicular infiltration followed by the disruption of the follicular unit.

Furthermore, the immunohistochemistry studies revealed increased production of proinflammatory molecules, like tumor necrosis factor- (TNF-) *α*, interleukin- (IL-) 1b, IL-12, IL-17, and IL-23.

In addition, TNF-*α* is one of the main cytokines that can be associated with the development of insulin resistance and hyperlipidemia, characteristics associated with metabolic syndrome.

Recent works suggest that HS should be viewed as a systemic inflammatory disease linked most commonly to metabolic, gastrointestinal, rheumatologic, psychiatric, and cardiovascular (CV) comorbidities.

The prevalence of the sympathetic branch of the autonomous cardiovascular system in the population reflects an increased CV risk. In this study, we used Heart Rate Variability (HRV) as a noninvasive tool to assess the sympathetic-vagal equilibrium in the autonomous CV regulation system [8–10]. Our aim was to evaluate whether autonomic function is affected in young HS patients and whether to consider HS as an independent cardiovascular risk factor [11].

## 2. Material and Methods

### 2.1. Subjects

Subjects are those affected by moderate to severe HS who attended our Outpatient Service of Dermatology from October 2018 to April 2019.

The heart rate (HR), RR trait (in ms), PQ interval (v.n.: 120-220 ms), QRS (v.n.: <100 ms), and QTc (v.n.: <430 ms) were measured.

Patients were informed not to assume any modifiers of autonomic nervous system functions such as psychoactive drugs and caffeine within 4 hours prior to the electrocardiogram (ECG). Informed consent was collected from each patient.

### 2.2. Assessments

We used the Cardiolab CE pocket PC ECG system in order to obtain an 8 min digital ECG at rest in supine position and during 8 minutes of orthostatic position obtained by the use of a passive tilt table.

With dedicated software (Cardiolab—Xai Medica), we analyzed the HRV indexes in the time and frequency domains. We will also analyze the nonlinear dynamics of HRV by Kubios software [12].

### 2.3. Linear Methods

We used linear methods in order to obtain a direct estimation of HRV through two types of analysis: time-domain analysis and frequency-domain analysis.

The time-domain method is based on NN intervals which are related to RR intervals in the ECG. They are analyzed to acquire variables such as SDNN (Standard Deviation of all NN intervals) and RMSSD (Root Mean Square of Successive Differences between adjacent NNs); both indexes represent parasympathetic tone.

Frequency-domain analysis, which is based on the power spectral density of the heart rate time series, highlights the issue of the underlying rhythms of the mechanisms controlling the heart rate (HR) and identified three major spectral peaks: high frequency (HF: 0.15-0.4 Hz), low frequency (LF: 0.04-0.15 Hz), and very low frequency (VLF: below 0.04 Hz) in the adult HR spectrum.

LF is an indicator of both sympathetic and parasympathetic activities. HF reflects parasympathetic activity. The LF/HF ratio is an index representing overall balance between sympathetic and parasympathetic systems. Higher values reflect dominance of the sympathetic branch, while lower ones reflect the predominance of the parasympathetic action.

### 2.4. Nonlinear Methods

The nonlinear methods (Poincarè plots and Detrended fluctuation analysis) are not modified by environmental features and measure the complex mechanisms regulating the signal [13].

**Figure 1:**
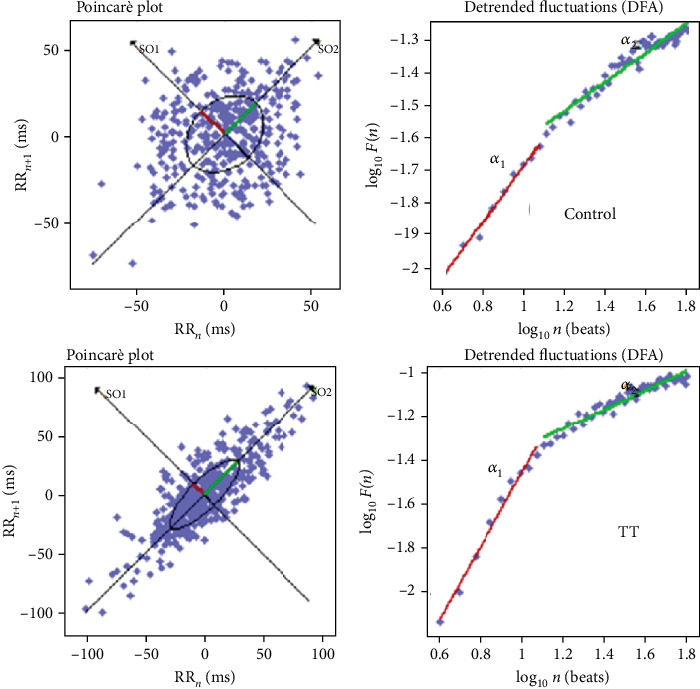
An example of Poincarè plot and DFA during a Tilt Test (TT) in a patient with HS.

Poincarè plots is a two-dimensional vector analysis that was used to quantify the shape of the plots (Figure 1). In this quantitative method, short-term (SD1) and long-term (SD2) RR interval variability and the ellipse area of the plot are quantified separately. SD1 is an indicator of vagal modulation of the sinus node. The HR correlations were defined separately for short-term (<11 beats, *α*1) and longer-term (>11 beats, *α*2) RR interval data. The *α*1 index was positively correlated with LF in normalized units. The other nonlinear method is the detrended fluctuation analysis (DFA) that consists of a procedure utilized to measure the fractal scaling assets of short-term and intermediate-term variability in the RR ECG intervals. The decrease of the *α*1 index is considered a mortality prognostic factor in patients affected by severe cardiovascular conditions such as ischemic events and cardiac insufficiency.

### 2.5. Data Analysis

Our study results were examined through two types of software: Cardiolab, Xai Medica (used to investigate HRV linear methods), and Kubios HRV 2.0 (for the HRV nonlinear analysis examination). We expressed our data as the mean ± standard error. We calculated the *t*-test in order to estimate the quantitative variables, and the chi-squared test was performed to estimate the qualitative ones.

We performed a statistical analysis using the software SigmaStat 3.5 (Systat Software Inc., Point Richmond, CA, USA). We considered a *P* value with a significance level < 0.05.

## 3. Results

Sixteen HS patients were recruited, including 9 females and 7 males aged between 16 and 48 years. Four of sixteen patients have normal weight, 6/16 were overweight, 3/16 patients presented with grade I obesity, 2/16 patients presented grade II obesity, and one patient presented with grade III obesity. In addition, eight of sixteen patients were smokers.

Information about gender, age, weight, height, BMI, and smoking habits is reported in Table 1.

We did not observe any significative difference when we catalogued patients according to BMI, though an increase of the heart rate (RR) was observed in subjects with a BMI > 25 (Table 2).

On the difficulty of having clean data, we analyzed 8/16 patients in detail, as preliminary data: 3 males and 5 females aged between 16 and 48 years. Of these subjects, only 2 patients presented with a normal weight while 6 patients had a BMI greater than 24.99 kg/m^2^ (2 patients were overweight, 3 patients had a grade I obesity, and 1 patient had a grade III obesity). Five patients were smokers, and 3 patients were nonsmokers. In all patients, the age of onset was between 10 and 28 years while the age of diagnosis was between 15 and 45 years. The ECG recorded did not show any alteration, except in one patient, where it showed a right conduction disorder (Table 3).

As expected, from the supine to orthostatic position, RR interval showed a significant decrease (from 796.3 ± 110.8 to 674.68 ± 56.95 msec—*P* < 0.001); systolic and diastolic arterial pressure showed a slight increase as in normal subjects (Tables 4 and 5).

We also compared the HRV indexes of subjects with HS with those observed in a group of 14 normal subjects from our previous work. We observed a reduction of total HRV in the time and frequency domain: the STD showed a significant decrease in HS patients (40.32 ± 5.79 to 33.1 ± 19.4 msec). The sympathetic indexes in HS patients are higher than those in normal subjects even if not all of them reached statistical significance (LF/HF ratio 1.13 ± 0.24 vs. 1.42 ± 1.1, DFA *α*1 0.85 ± 006 vs. 1.04 ± 0.29).

## 4. Discussion

HS is an inflammatory skin disease of the terminal hair follicle. It manifests clinically with the onset of painful nodules, abscesses, and draining sinus tracts eventually resulting in scars, involving multiple regions of the body.

Some recent studies have suggested a connection between HS and a significantly increased risk of ischemic cardiovascular events, such as myocardial infarction and stroke. It was also suggested that the risk of ischemic events is higher in subjects affected by HS when compared to severe psoriasis patients [14, 15]. The association between inflammatory markers and sympathetic hyperactivation indexes remained significant after correcting important risk factors and confounding factors such as age, Body Mass Index, heart rate, and smoking. Sympathetic activation can also be triggered by reflex mechanisms (arterial baroreceptor impairment), psychological stress, oxidative stress, obstructive sleep apnea, inflammation, and metabolic factors as I.R. and dysregulated production and secretion of adipokines from visceral fat with a particular important role of leptin [16].

Interestingly, a Danish cross-sectional study showed that the mean heart rate in severe HS patients in resting conditions was suggestively higher when compared with that of controls [17]. Both HS and atherosclerosis have a chronic inflammatory subset, and cardiovascular and metabolic diseases are the most common comorbidities found in HS [18, 19]. Atherosclerosis, a chronic inflammatory disease affecting the blood vessels, is considered a major cause of cardiovascular diseases. Markers of inflammation are associated with both cardiovascular risk and the severity of HS inflammatory manifestations. It is known that inflammation represents the core in the bridge between HS and the pathogenesis of cardiovascular diseases, though many doubts remain regarding its mechanism of action. An important role is played by interleukin- (IL-) 17, which is found to be upregulated in lesional and perilesional skin of HS patients [20]. Overexpression of various other cytokines, such as IL-1, TNF-*α*, IL-10, and IL-11, as well as antimicrobial peptides (AMPs), beta-defensin 2, psoriasin, and cathelicidin, has been observed in the lesional setting.

Interleukin- (IL-) 32 expression is significantly enhanced in plaque of atherosclerosis, and its proinflammatory function could help increase the risk of CV events in patients affected by chronic inflammatory diseases [21, 22]. Moreover, HS patients generally present a higher incidence of other cardiovascular risk factors such as obesity, smoking history, diabetes, hypertriglyceridemia, and metabolic syndrome [23].

The present study was based on the analysis of HRV as a noninvasive tool to evaluate the association between HS and an increased CV risk. The mechanism leading to depressed HRV in heart failure is complex and not perfectly described, but it could partially be related to the alteration of neurohumoral activity [24].

The results indicate that the sympathetic activity increases during the Tilt Test more than in normal subjects as shown by the tachycardia and by the increase of the arterial pressure and LF/HF ratio and the decrease of the STD. The Tilt Test induces, also, an increase in the properties of short-term fractal correlations of heart rate dynamics (DFA) accompanied with a decrease in all the nonlinear indexes of HRV, confirming the thesis that these indexes are an expression of the cardiac sympathetic-vagal balance.

HS patients, if compared to the rest of the population, tend to present a higher incidence of carotid atherosclerosis. The role of smoking in the severity of HS is still not clear because it is not known whether smoking cessation improves the course of the disease.

A higher resting heart rate and a lower heart rate variability are linked to subclinical chronic inflammation in adult and elderly patients. The higher death rate that was described in this kind of situations could therefore manifest a shared etiology. A disproportion of the sympathetic branch of the autonomic nervous system could correlate with inflammatory events to play a major role in atherosclerosis.

It has been previously demonstrated that the use of HRV allows us to evidence an upregulated CV risk in other inflammatory dermatological disorders, such as psoriasis [25]. As reported in patients with psoriasis, in patients with HS, the sympathetic activation indexes (LF/HF, SD2/SD1, and *α*1/*α*2) are higher than those in normal subjects too.

Studies conducted on patients with known HS indicate that many of them are overweight. Obesity is one of the main risk factors associated with the development of HS, although its role is yet to be defined. We did not observe any significant difference when reorganizing patients according to the BMI, although an increase of heart rate (RR) was observed in subjects with a Body Mass Index (BMI) > 25. Furthermore, we observed that the sympathetic activation indexes during passive orthostatism had increased compared to clinostatism.

## 5. Conclusions

Our data show a balanced reduction of the parasympathetic influence on the sinus node in patients with moderate to severe HS. These results could correlate with an increased risk of cardiovascular disease; thus, HS should be considered as an independent CV risk factor.

Our data seems to indicate an increase in the sympathetic hyperactivity indices in HS patients, but since our study was based on a limited number of cases, we consider it to be preliminary and more studies are necessary in order to obtain statistical significance. Identification of individuals at risk for subsequent morbidity and nonrisk groups requires future prospective studies to determine the sensitivity, specificity, and predictive values of HRV.

Moreover, our data demonstrates that biological therapy and other anti-inflammatory therapies could alter the autonomous cardiac function in patients with moderate to severe HS, as reported in a previous work on psoriasis [26]. The important connection between HS and cardiovascular diseases underlines the necessity of a cardiovascular screening in HS patients, particularly if other risk factors are present.

Although the exact role of smoking in the pathogenesis of HS remains to be determined, HS could give us another opportunity to encourage our patients to change their lifestyle.

Dermatologists must be conscious of the comorbidities related to HS. The aim is to create a condition in which patients are managed by the proper specialists. This may lead to an improvement in the disease course and in the quality of life of patients affected by this disease.

## Figures and Tables

**Table 1:** HS patients included in the study and assessed with ECG.

Age (years)	Sex	Height (cm)	Weight (kg)	BMI	Smoke	HR (bpm)	RR (ms)	PQ (ms)	QRS (ms)	QTc (ms)
41	F	1.68	83	29.4	Yes	78	768	114	88	324.00
16	F	1.65	72	26.4	No	93	642	148	58	296.00
31	M	1.7	95	32.9	Yes	81	740	140	92	344.00
15	M	1.62	93	35.4	No	87	690	140	78	332.00
48	M	1.8	90	27.8	Yes	60	1004	170	84	410.00
13	F	1.42	79	39.2	No	89	672	134	80	303.00
28	F	1.7	85	29.4	Yes	74	816	98	70	361.00
16	M	1.85	85	24.8	No	59	1020	152	88	404.00
36	M	1.66	77	27.9	No	70	856	148	88	370.00
19	F	1.76	72	23.2	No	78	768	122	76	324.00
32	F	1.65	83	30.5	Yes	79	758	148	86	322.00
28	F	1.57	76	30.8	Yes	67	892	144	84	349.00
24	F	1.7	130	45	Yes	93	644	156	88	297.00
16	F	1.67	60	21.5	No	83	720	172	108	314.00
22	M	1.85	95	27.8	Yes	74	806	104	110	359.00
21	M	70	1.85	20.5	No	69	866	120	88	372.00

25.38		6.23	79.80	29.53		77.125	791.375	138.125	85.375	342

**Table:** (a) Normal weight

Physical condition	Smoke	HR	RR	PQ	QRS	QTc
Normal weight	No	59	1020	152	88	404.00
Normal weight	No	78	768	122	76	324.00
Normal weight	No	83	720	172	108	314.00
Normal weight	No	69	866	120	88	372.00
		72.25	843.5	141.5	90	353.50

**Table:** (b) Overweight and obesity

Physical condition	Smoke	HR	RR	PQ	QRS	QTc
Obesity I	Yes	81	740	140	92	344.00
Obesity I	Yes	79	758	148	86	322.00
Obesity I	Yes	67	892	144.0	84	349.00
Obesity II	No	87	690	140	78	332.00
Obesity II	No	89	672	134	80	303.00
Obesity III	Yes	93	644	156	88	297.00
Overweight	Yes	78	768	114	88	324
Overweight	No	93.0	642	148	58	296.00
Overweight	Yes	60	1004	170	84	401.00
Overweight	Yes	74	816	98	70	361.00
Overweight	No	70	856	148	88	370.00
Overweight	Yes	74	806	104	110	359.00
		78.75	774	137	85.38	338.17

**Table 3:** Characteristics of HS patients evaluated with HRV (Heart Rate Variability).

Age	Weight (kg)	Height (m)	BMI	Physical condition	Smoke	Age of onset	Age of diagnosis	ECG
16	72	1.65	26.4	Overweight	No	10	15	Normal
31	95	1.7	32.9	Obesity I	Yes	20	30	Normal
48	90	1.8	27.8	Overweight	Yes	20	45	dx conduction disorder
19	72	1.76	23.2	Normoweight	No	14	18	Normal
32	83	1.65	30.5	Obesity I	Yes	28	28	Normal
28	76	1.57	30.8	Obesity I	Yes	15	25	Normal
24	130	1.7	45	Obesity III	Yes	13	23	Normal
21	70	1.85	20.5	Normoweight	No	14	20	Normal

**Table 4:** HRV values obtained using both linear and nonlinear methods in clinostatic position.

Clino	RR (ms)	STD RR (ms)	LF (%)	HF (%)	LF/HF (-)	SD₁ (ms)	SD₂ (ms)	SD₂/SD₁	*α*₁	*α*₂	*α*₁/*α*₂
	675	24.5	71	23.2	3.06	12.6	32.3	0.39	1.411	0.302	4.67
	757.1	16.9	67.3	23.8	2.83	11.5	20.9	0.55	1.272	0.46	2.77
	965.5	34.3	33.3	62.1	0.54	33.3	35.4	0.94	0.627	0.372	1.69
	795.8	36.6	40.9	51.5	0.79	27.9	43.7	0.64	0.839	0.436	1.92
	766.2	26.1	38.8	49.7	0.78	19.1	31.7	0.6	1.015	0.443	2.29
	860.6	42.3	29.6	62.3	0.48	19.6	28.5	0.69	0.69	0.39	1.77
	642.8	10.4	55.6	31	1.79	7	12.9	0.54	1.024	0.525	1.95
	907.6	73.6	35.9	61.4	0.58	57.1	87.1	0.66	0.829	0.203	4.08

Mean	796.3	33.1	46.6	45.6	1.4	23.5	36.6	0.6	1	0.4	2.6
SD	110.8	19.4	15.9	17.1	1.1	16.1	22.4	0.2	0.3	0.1	1.1

**Table 5:** HRV values obtained using both linear and nonlinear methods in orthostatic position.

Ortho	RR (ms)	STD RR (ms)	LF (%)	HF (%)	LF/HF (-)	SD₁ (ms)	SD₂ (ms)	SD₁/SD₂	*α*₁	*α*₂	*α*₁/*α*₂
	599.5	27.9	79.1	7.5	10.55	9	38.4	0.23	1.714	0.486	3.53
	724.5	18.5	63.2	23.4	2.70	9.6	24.4	0.39	1.401	0.558	2.51
	746.7	30.3	57.6	34.4	1.67	25.8	34.2	0.75	0.975	0.373	2.61
	741.8	25.3	47.7	46.9	1.02	14.3	32.8	0.44	1.126	0.422	2.67
	669.8	20.2	63.9	20.7	3.09	11	26.3	0.42	1.299	0.63	2.06
	646.8	23.2	86	5.3	16.23	6.8	32.1	0.21	1.759	0.522	3.37
	614.3	13.7	54.6	36	1.52	7.6	17.8	0.43	1.080	0.576	1.88
	654	41.8	54.4	39.3	1.38	23.3	54.4	0.43	1.198	0.329	3.64

Mean	674.68	25.11	63.31	26.69	4.77	13.43	32.55	0.41	1.32	0.49	2.78
SD	56.95	8.58	13.07	15.05	5.57	7.26	10.94	0.16	0.29	0.10	0.66

## Data Availability

The authors are available to provide data supporting the research.
